# Exploring the Interplay of Socioeconomic and Behavioral Factors: Unraveling Gender Disparities in Glycemic Control Among Adult Type 2 Diabetic Patients in Outpatient Care

**DOI:** 10.7759/cureus.56505

**Published:** 2024-03-19

**Authors:** Amar Mankar, Umesh Kawalkar, Nilesh Jadhao, Umesh Joge, Ashutosh Paldiwal, Manoj Talapalliwar, Manoj S Patil

**Affiliations:** 1 Community Medicine, Government Medical College (GMC), Akola, IND; 2 Community Medicine, Shri Vasantrao Naik Government Medical College, Yavatmal, IND; 3 Pediatrics, Government Medical College (GMC), Akola, IND; 4 Research and Development, Datta Meghe Institute of Higher Education and Research, Wardha, IND

**Keywords:** socioeconomic, glycemic level, gender, management, t2dm

## Abstract

Background

Diabetes mellitus (DM) presents global challenges, with optimal glycemic control being pivotal in managing complications, notably in type 2 diabetes mellitus (T2DM). Yet, achieving sustained control faces barriers stemming from socioeconomic and gender-specific disparities. This study addresses these gaps by examining socioeconomic determinants and gender disparities in diabetes management, particularly in Maharashtra, India.

Methodology

This cross-sectional study involved 302 T2DM patients aged 20 to 79 years. Data on sociodemographic, behavioral, and clinical factors were collected through interviews, and records were analyzed via logistic regression to identify predictors of glycemic control.

Results

Significant associations emerged between gender and education, occupation, and religion. Glycemic control, with a mean HbA1c of 8.45%, remained suboptimal. Logistic regression identified gender, average family income, diabetes duration, treatment nature, comorbidities, complications, and medication adherence as glycemic control predictors.

Conclusions

Addressing socioeconomic and gender-specific factors is paramount in diabetes management, especially in rural areas where sociocultural influences shape health behaviors. Tailored interventions, including gender-sensitive health education, are vital for improving diabetes care and outcomes. This study provides crucial insights into gender-specific influences on glycemic control among T2DM patients in Maharashtra, advocating for personalized interventions to enhance overall diabetes management.

## Introduction

Diabetes mellitus (DM) is a chronic noncommunicable disease (NCD) characterized by persistent high blood glucose levels in which the body cannot make enough insulin or use it efficiently [1]. Diabetes affects individuals, families, and the community as a whole in terms of lost employment, poor quality of life, frequent emergency department visits, hospitalizations, and mortality [2]. Maintaining adequate glycemic control throughout the disease is the sole practical method to reduce complications from diabetes [3]. Patients with type 2 diabetes mellitus (T2DM) with inadequate glycemic control are at a high risk of developing diabetic complications, which represents a severe public health concern [4]. In clinical practice, attaining optimum long-term glycemic control is challenging because the reasons for poor glycemic control in type 2 diabetes are diverse [5]. The management of T2DM extends beyond the confines of medical interventions, delving into the intricate web of socioeconomic and behavioral determinants that significantly influence glycemic control [6].

Several studies conducted globally and within India have investigated the social determinants of health (SDHs) and gender-specific disparities in glycemic control among adults with type 2 diabetes [7]. The existing literature presents conflicting views on the influence of gender differences in achieving and maintaining optimal glycemic control [8]. In a study that included a global perspective, socioeconomic determinants and gender disparities in glycemic control among adult T2DM patients were explored [9]. The research highlighted the impact of factors such as income, educational attainment, and occupational status on access to healthcare resources, treatment adherence, and the adoption of diabetes-friendly lifestyles [9]. Additionally, a study specifically investigated glycemic control in the Indian context, shedding light on the socioeconomic and behavioral factors affecting male and female individuals managing type 2 diabetes in India [10]. The research provided valuable insights into the unique challenges faced by the Indian population [10].

Understanding and addressing gender-specific differences within socioeconomic determinants and behavioral factors are critical for tailoring interventions to the unique challenges faced by both male and female individuals managing type 2 diabetes, particularly in diverse cultural contexts like India [11].

The National Programme for Prevention and Control of Cancer, Diabetes, Cardiovascular Diseases, and Stroke, launched in 2010, has been a crucial initiative in India. However, a gap exists within the program, as it does not adequately consider SDHs or gender-specific factors [12]. Our study addresses this limitation, seeking to bridge the gap by integrating an examination of SDHs and gender disparities into the discourse surrounding diabetes management. Through this, we aim to contribute to developing targeted and gender-sensitive strategies, ultimately improving the overall health and well-being of adult type 2 diabetic patients in India.

This study aims to fill a notable gap in the existing research by emphasizing the need to comprehensively examine gender-related factors influencing glycemic control outcomes among this population. Limited studies have specifically investigated the interplay between gender and NCDs, including type 2 diabetes (World Health Organization, 2018). Our research seeks to contribute valuable insights into this underexplored area by critically examining socioeconomic and behavioral factors. Furthermore, our study recognizes the unique risks men and women face, especially in rural areas. For instance, rural women may encounter distinct challenges related to socioeconomic disparities, limited access to healthcare resources, and traditional gender roles, all of which could elevate their risk of diabetes. Our research aims to shed light on these specific factors, providing a nuanced understanding of the gender-related risks associated with diabetes, particularly in rural contexts.

## Materials and methods

Study setting and participants

This cross-sectional study was conducted at the Department of Community Medicine in Government Medical College, Akola (Maharashtra). The study included participants diagnosed with type 2 diabetes, aged between 20 and 79 years, attending outpatient services with a confirmed diagnosis documented in treatment records, and undergoing treatment for more than one year.

Sample size determination

The calculation of the sample size was based on the study by Haghighatpanah et al. in a South Indian teaching hospital, Manipal, India, where 78.2% of the study population had poor glycemic control [4]. With a 95% confidence interval (CI), a 5% acceptable margin of error, and a design effect of 1, the minimum required sample size was calculated as 262 using OpenEPI Software. Considering a nonresponse rate of 15%, a total of 302 study samples were included. Samples were recruited using convenience sampling.

Ethical considerations

Ethical clearance was obtained from the Institutional Ethics Committee, Government Medical College, Akola (reference number: 90/2022). Data were collected using patients’ case notes and semi-structured, interviewer-administered questionnaires. After providing all necessary details, participants provided their informed consent.

Data collection and variables

Data, including medical history, demographic information, HbA1c test values, and/or the latest two fasting blood glucose (FPG) test results, were extracted from available records and corroborated by patients. Trained data collectors utilized semi-structured, Marathi-language questionnaires to evaluate behavior and lifestyle, complications, treatment-related factors, and healthcare provider-associated variables.

Definition of poor glycemic control

Patients with an HbA1c of 7% or higher (or an average FPG of 126 mg/dL or higher when no HbA1c test was available) had poor glycemic control. The study population’s prevalence of both poor and good glycemic control was ascertained using this classification.

Data analysis

Epi Info was used for descriptive analyses, producing mean, median, standard deviation (SD), variance, and range for numerical data. Frequency tables were constructed for categorical variables, and some were converted into binary variables for relative measure calculations and analysis of the degree of correlation across different categories of glycemic control.

## Results

A total of 302 type 2 diabetics were questioned to examine the association between glycemic control and sociodemographic background, behavior, and treatment modality. Univariate analysis showed that religion, education status, and occupation were significantly associated with gender distribution. Other variables such as age, marital status, religion, and average income were not significant (Table 1).

**Table 1:** Sociodemographic characteristics of the study participants. Chi-square test.

Variable	Male (n = 177)		Female (n = 125)		P-value
	Frequency	Percentage	Frequency	Percentage	
Age (years)					
Mean age	53.3	9.8	52.3	10.1	0.371
Marital status					
Living with spouse	154	87%	101	80.80%	0.143
Living without spouse	23	13%	24	19.20%	
Residence					
Rural	83	46.90%	55	44%	0.619
Urban	94	53.10%	70	56%	
Religion					
Hindu	136	76.80%	84	67.20%	0.05*
Muslim	11	6.20%	19	15.20%	
Buddhist	24	13.60%	16	12.80%	
Other	6	3.40%	6	4.80%	
Education status					
Illiterate	8	4.50%	16	12.80%	0.003*
Primary level	59	33.40%	55	44%	
Higher secondary level	48	27.1	27	21.60%	
Graduate and above	62	35%	27	21.60%	
Occupation					
Unemployed	42	23.80%	26	20.80%	0.000*
Government servant	25	14.10%	8	6.40%	
Non-government servant	19	10.70%	4	3.20%	
Business/self-employed	79	44.60%	11	8.80%	
Retired	12	6.80%	4	3.20%	
Housewife	0	0	72	57.60%	
Average family income					
>50,000	82	46.30%	71	56.80%	0.199
30,000–50,000	45	25.40%	25	20%	
<30,000	50	28.30%	29	23.20%	

The patients’ glycemic control level was evaluated using HbA1c and FPG. Prioritizing HbA1c, the globally advised gold standard for tracking glycemic control, allowed for a more accurate evaluation of the patients’ blood glucose control. Although most diabetic patients in low- and middle-income nations, such as India, do not often have access to the HbA1c test. According to the American Diabetes Association’s diabetes guideline, an HbA1c of less than 7% was considered satisfactory glycemic control for this patient population. In our study, 190 participants had HbA1c values assessed within six months. The respondents’ HbA1c ranged from 5.2% to 18%, with a mean (SD) of 8.45% (0.77%). For the rest of the participants (n = 112) who had no recent HbA1c test results, the average of their latest FPG readings was used to determine their level of glycemic control. Patients were classified as having reasonable glycemic control if their average FPG was less than 126 mg/dL and poor glycemic control if their average FPG was equal to or greater than 126 mg/dL. Our study cohort had an average (SD) FPG of 156.6 (55.6), ranging from 71 mg/dL to 350 mg/dL (Table 2).

**Table 2:** Gender-wise distribution of diabetes-related variables. Chi-square test.

Variable	Male (n = 177)		Female (n = 125)		P-value
	Frequency	Percentage	Frequency	Percentage	
Family history					
Yes	70	39.50%	46	36.80%	0.629
No	107	60.50%	79	63.20%	
Duration of diabetes					
<7 years	84	47.50%	70	56%	0.199
>7 years	93	52.50%	55	44%	
Glycemic control					
Yes	32	18%	33	26.40%	0.083
No	145	82%	92	73.60%	
Nature of diabetes mellitus treatment					
Only oral medications	108	61%	72	57.60%	0.436
Only insulin	13	7.30%	6	4.80%	
Combination of insulin and oral medications	56	31.70%	47	37.60%	
Comorbidity					
Yes	96	54.20%	77	61.60%	0.203
No	81	45.80%	48	38.40%	
Diabetic complications					
Yes	82	46.30%	56	44.80%	0.793
No	95	53.70%	69	55.20%	
Forgetting to take medication					
Never	40	22.60%	23	18.40%	0.083
Seldom (once per month)	97	54.80%	71	56.80%	
Sometimes (once per week)	21	11.90%	17	13.60%	
Often (more than once per week)	19	10.70%	14	11.20%	

All diabetes-related factors, i.e., family history, duration of diabetes, poor glycemic control, nature of diabetes treatment, comorbidities, diabetic complications, and forgetting to take medications, did not have statistically significant gender-wise distribution. Behavior and lifestyle variables among study participants were not statistically substantial for gender-wise distribution except for tobacco and alcohol consumption (Table 3).

**Table 3:** Gender-wise distribution behavior and lifestyle variables of the study participants. Chi-square test.

Variable	Male (n = 177)		Female (n = 125)		P-value
	Frequency	Percentage	Frequency	Percentage	
Adherence to at least 30 minutes of physical activity (total minutes of continuous activity including walking) in weeks					
Good adherence (6–7 days/weeks)	85	48%	59	47.20%	0.768
Moderate adherence (4–5 days/week)	31	17.50%	23	18.40%	
Poor adherence (2–3 days/week)	29	16.50%	16	12.80%	
Non-adherence (0–1 day/week)	32	18%	27	21.60%	
Adherence to a healthy eating plan in weeks					
Good adherence (6–7 days/weeks)	79	44.60%	60	48%	0.702
Moderate adherence (4–5 days/week)	34	19.20%	23	18.40%	
Poor adherence (2–3 days/week)	46	26%	33	26.40%	
Non-adherence (0–1 day/week)	18	10.20%	9	7.20%	
Frequency of daily consumption of fruits and vegetables in weeks					
Regular (6–7 days/week)	45	25.40%	33	26.40%	0.99
Often (4–5 days/week)	34	19.20%	23	18.40%	
Sometimes (2–3 days/week)	45	25.40%	33	26.40%	
Seldom (0–1 day/week)	53	30%	36	28.80%	
On how many of the last seven days did you eat high-fat foods such as fried food (e.g., samosa), red meats, and dairy foods?					
Regular (6–7 days/week)	7	4%	4	3.20%	0.197
Often (4–5 days/week)	23	13%	8	6.40%	
Sometimes (2–3 days/week)	46	26%	29	23.20%	
Seldom (0–1 day/week)	101	57%	84	67.20%	
On how many of the last seven days did you consume sugar and sugar-containing foods (desserts) and soft drinks?					
Regular (6–7 days/week)	11	6.20%	6	4.80%	0.99
Often (4–5 days/week)	14	8%	8	6.40%	
Sometimes (2–3 days/week)	38	21.40%	26	20.80%	
Seldom (0–1 day/week)	114	64.40%	85	68%	
On how many of the last seven days did you check your blood sugar?					
Regular (6–7 days/week)	36	20.30%	24	19.20%	0.08
Often (4–5 days/week)	14	8%	6	4.80%	
Sometimes (2–3 days/week)	32	18%	12	9.60%	
Seldom (0–1 day/week)	95	53.70%	83	66.40%	
On how many of the last seven days did you check the insides of your shoes?					
Regular (6–7 days/week)	23	13%	19	15.20%	0.522
Often (4–5 days/week)	13	7.30%	5	4%	
Sometimes (2–3 days/week)	17	9.60%	9	7.20%	
Seldom (0–1 day/week)	124	70.10%	92	73.60%	
Tobacco consumption					
Yes	21	11.90%	0	0	0
No	156	88.10%	125	100%	
Alcohol consumption					
Yes	18	10.20%	0	0	0
No	159	89.80%	125	125	

To predict the outcome of glycemic control, a logistic model was fitted using predictor variables such as age, marital status, place of residence, religion, level of education, occupation, average family income, family history, length of diabetes, type of diabetes treatment, comorbidity, and medication forgetfulness. The model’s explanatory power was high (Tjur’s R2 = 0.37). Age ≥25 years corresponded to the model’s intercept at -5.27 (95% CI [-6.30, -4.35], p < 0.001). Standardized parameters were produced by fitting the model on a standardized version of the dataset. P-values and 95% CIs were calculated with the Wald z-distribution approximation (Table 4).

**Table 4:** Predictor variables for glycemic control.

Predictors	Sig.	Odds ratio	95% CI for odds ratio	
Gender	0.031	0.202	0.047	0.866
Average family income	0.005	0.259	0.100	0.671
Duration of diabetes	0.021	6.197	1.313	29.251
Nature of treatment	0.000	0.182	0.095	0.352
Comorbidity	0.020	4.053	1.249	13.149
Diabetic complication	0.001	0.116	0.033	0.405
Forgot to take medication	0.003	2.986	1.455	6.127
Regular physical activity (6–7 days/week)	0.007	0.008	0.000	0.256
Often physical activity (4–5 days/week)	0.005	0.006	0.000	0.214
Regular consumption of fruits (6–7 days/week)	0.050	7.826	1.001	61.208
Often consumption of fruits (4–5 days/week)	0.001	4.920	4.838	50.049
Constant	0.042	92108.308	0	0

The effect of gender, average family income, duration of diabetes, nature of the treatment, comorbidities, diabetic complications, forgetting to take medications, regular and frequent physical activity, and regular and frequent consumption of fruits were statistically significant.

## Discussion

DM is a pervasive global health challenge with profound implications for individual health and public well-being. Achieving and maintaining optimal glycemic control is imperative to mitigate the risk of diabetic complications. Our study critically examines the intricate web of socioeconomic and behavioral factors, unraveling potential gender disparities in glycemic control among adult T2DM patients.

The literature on gender differences in glycemic control has presented conflicting views, prompting our investigation into this critical area. Socioeconomic factors such as income, education, and occupation are pivotal in determining access to healthcare resources and adherence to treatment regimens. Recognizing potential gender disparities within these factors is essential for tailoring interventions to the unique challenges faced by male and female individuals managing type 2 diabetes. Moreover, behavioral factors, including lifestyle choices, dietary habits, physical activity, and medication adherence, contribute significantly to glycemic control. An in-depth exploration of these factors, stratified by gender, provides a nuanced perspective on the challenges and opportunities for intervention. Our cross-sectional study, conducted at a government medical college in Maharashtra comprising 302 type 2 diabetic patients aged 20-79 years, aimed to determine whether gender differences impact glycemic control outcomes, considering socioeconomic and behavioral factors. The study revealed several significant findings that contribute to the broader understanding of diabetes management.

The sociodemographic characteristics of the study participants showed significant associations between gender and education, occupation, and religion. These findings are consistent with the research conducted by Sujata and Thakur, which analyzed National Family Health Survey data and observed that Christian and Muslim adults were most affected by diabetes, potentially due to lifestyle and eating habits [13]. The study analyzed diabetes-related variables and found no statistically significant gender differences in family history, duration of diabetes, nature of the treatment, comorbidities, diabetic complications, or medication adherence. These findings align with research conducted by Borgharkar and Das, which demonstrated real-world evidence of glycemic control among patients with T2DM in India [10]. Furthermore, Borgharkar and Das identified factors such as obesity, hypertension, and diabetes duration as statistically associated with uncontrolled glycemia, emphasizing the multifactorial nature of diabetes management.

Glycemic control, assessed through HbA1c and FPG levels, revealed an overall mean HbA1c of 8.45%, indicating suboptimal control. The logistic regression model identified several predictors significantly associated with glycemic control, including gender, average family income, duration of diabetes, nature of the treatment, comorbidities, diabetic complications, and medication adherence. These results are in line with studies emphasizing the influence of socioeconomic and behavioral factors on glycemic control [10,14,15]. Behavioral and lifestyle variables, stratified by gender, demonstrated no significant differences in physical activity adherence, healthful eating, or consumption of fruits and vegetables. However, tobacco and alcohol consumption showed substantial gender disparities. These findings align with studies indicating gender-specific patterns in dietary practices and self-efficacy related to diabetes care [14,15].

Comparing our results with existing literature, we note similarities and differences. Distinct variations were identified within the social and cultural fabric, dietary habits, healthcare distribution, and women’s traditional roles in rural India. This study aligns with global trends in diabetes prevalence and socioeconomic influences on disease management. However, the unique sociocultural context may contribute to variations in healthcare utilization patterns and dietary practices, emphasizing the importance of context-specific interventions. This approach is vital because it acknowledges that diverse communities may face distinct challenges and barriers in adhering to recommended health behaviors. Regarding healthcare utilization, different sociocultural backgrounds may influence how individuals seek and engage with medical services, emphasizing the need for tailored healthcare approaches. There is a need to develop gender-specific health education programs to address the rural population, specifically women.

Limitations

This study has limitations. Cross-sectional data impedes the establishment of causality. Potential sampling bias might restrict the generalizability of results. Reliance on self-reported data introduces potential biases such as recall and social desirability.

## Conclusions

Our study provides valuable insights into the gender-specific nuances of socioeconomic and behavioral factors influencing glycemic control among type 2 diabetic patients. The identification of gender as a significant predictor highlights the need for targeted, gender-sensitive interventions to enhance diabetes management. These findings contribute to the ongoing dialogue on personalized approaches to diabetes care, with implications for healthcare policies, interventions, and future research in this field.
